# Remineralization and protection from demineralization: effects of a hydroxyapatite-containing, a fluoride-containing and a fluoride- and hydroxyapatite-free toothpaste on human enamel in vitro

**DOI:** 10.1186/s13005-022-00330-5

**Published:** 2022-07-13

**Authors:** Leona Guntermann, Arno Rohrbach, Edgar Schäfer, Till Dammaschke

**Affiliations:** 1 Private Practice, Westfälische Str. 18, 57462 Olpe, Germany; 2 Institute of Mineralogy, Westphalian Wilhelms-University, Corrensstr. 24, 48149 Münster, Germany; 3 Central Interdisciplinary Ambulance in the School of Dentistry, Westphalian Wilhelms-University, Waldeyerstr. 30, 48149 Münster, Germany; 4 Department of Periodontology and Operative Dentistry, Westphalian Wilhelms-University, Waldeyerstr. 30, 48149 Münster, Germany

**Keywords:** Fluoride, Hydroxyapatite, Remineralisation, Toothpaste

## Abstract

**Background:**

The aim was to evaluate the remineralization potential as well as the extent of protection against renewed demineralization of enamel by hydroxyapatite-containing toothpaste (Karex) in comparison to fluoride-containing (Elmex) and fluoride- and hydroxyapatite-free toothpaste (Ajona) as control.

**Methods:**

Fifty-seven enamel samples were obtained from 19 human teeth. Five demarcated surfaces were created on each tooth (S0—S4). Four of the surfaces (S1—S4) were exposed to lactic acid (pH 3) for 8 h (demineralization). S0 was left untreated as control. S1 was solely treated with acid. After demineralization, S2 was exposed to Karex for 2 min, of which 15 s were brushing. S3 was treated with Elmex and S4 with Ajona, accordingly. Then, the samples were evaluated using a scanning electron microscope and ImageJ image analysis software to determine the percentage of demineralization. Afterwards, S2-S4 were again exposed to lactic acid for 2 h, and subjected to pixel analysis another time. Data were statistically analysed using ANOVA with post-hoc Scheffé test and the Kurskal-Wallis test.

**Results:**

The surfaces treated with Elmex showed the lowest percentage of demineralization (mean 5.01 ± 0.98%) (*p* < 0.01). Thus, Elmex remineralized more effectively compared to Ajona (8.89 ± 1.41%) and Karex (9.85 ± 1.63%) (*p* < 0.01). Furthermore, Elmex showed the lowest percentage of demineralized enamel after new demineralization (median 6.29%), followed by Ajona (11.92%) and Karex (13.46%) (*p* < 0.001).

**Conclusion:**

In terms of remineralization and protection against renewed demineralization, a hydroxyapatite-containing toothpaste (Karex) appears to be inferior to a fluoride-containing toothpaste (Elmex) and a fluoride- and hydroxyapatite-free toothpaste (Ajona). Hence, the recommendation to use Karex to protect against demineralization should be critically questioned.

## Background

Fluoride is an active ingredient in toothpastes that has been extensively investigated in dentistry. Its effects in the oral cavity, on enamel surfaces and in the entire human organism are well known. Fluoride promotes remineralization and thus the incorporation of calcium and phosphate ions into the tooth enamel. Fluoride ions are themselves incorporated into the enamel and protect against demineralization, as the acid solubility of enamel decreases due to the incorporation of fluoride ions. The original relatively acid-soluble hydroxyapatite (HAP) is converted into relatively acid-stable fluorapatite. Furthermore, a covering calcium fluoride layer is formed on the tooth surface, which can serve as a “sacrificial layer” during future acid attacks [1–5]. After fluoridation, a formation of calcium hydroxide was observed between the superficial calcium fluoride layer and the enamel, consisting of fluorapatite [1]. Therefore, regular use of fluoride-containing oral health care products effectively protects teeth from bacterial-induced acid lesions causing dental caries [2–5]. In addition, fluoride application increases the abrasion resistance of enamel and protects teeth from erosive processes caused by acids from foods as well as beverages [6–8].

The success in caries prevention is based in particular on the wide availability of fluoride in toothpastes [9, 10]. Patients at risk of caries, such as the elderly or children of primary dentition age, can be particularly effectively protected from caries by increased fluoride administration—for example, in the form of fluoride gels or varnishes [2, 3]. If the recommended dosage is observed, there are no concerns about the use of fluorides even for young children [11]. Taking into account the usual recommendations for use in dentistry, there is no toxicological risk for fluorides in toothpastes [12].

Despite the fact that it is evidence-based that fluorides are effective in the prevention of caries and erosions and are not toxic in the recommended concentrations [2–5], alternatives to fluorides in oral hygiene products are always sought. HAP represents one of the possible alternatives. HAP is a biocompatible calcium phosphate with a similar chemical composition to the apatite crystals of human enamel [13]. Various toothpastes with added HAP have been available for several years. Since there is a great chemical similarity of HAP with human enamel, the active ingredient HAP is supposed to adhere to the tooth surface during tooth brushing, thus forming a protective layer against bacteria and acids, and also repairing enamel areas attacked by acid [14]. However, little is known about the interaction of HAP with tooth structure. Similarly, whether HAP is an agent that can affect caries, erosion, or plaque formation is currently not fully understood [12]. In the literature, some studies can be found that were able to show comparable, positive effects of HAP and fluoride in terms of remineralization and caries prevention; however, the vast majority of studies were unable to demonstrate statistically significant better remineralization features of HAP compared to fluoride [14].

Since 2018, a new toothpaste containing HAP has been launched onto the German market: Karex (Dr. Kurt Wolff, Bielefeld, Germany). Karex is fluoride-free and contains 10% microcrystalline HAP as active ingredient (see Table 1). According to the manufacturer, Karex is said to be able to repair areas of tooth enamel demineralized by acid through HAP deposition. Karex should form a protective layer on tooth surfaces that releases calcium ions during acid attack, thus protecting the tooth from demineralization. However, these manufacturer claims have not yet been scientifically proven.

**Table 1:** The names, manufacturers and composition of the tested toothpastes

Product Names	Manufacturers	Composition
Karex	Dr. Kurt Wolff GmbH & Co. KG, Bielefeld, Germany	10% hydroxyapatite, aqua, hydrated silica, glycerin, xylitol, hydrogenated starch hydrolysate, silica, cellulose gum, sodium methyl cocoyl taurate, sodium sulfate, menthol, eucalyptol, 1,2-hexanediol, caprylyl glycol, sodium cocoyl glycinate, tetrapotassium pyrophosphate, phosphoric acid, zinc chloride, cetylpyridinium chloride
Elmex	CP GABA GmbH, Hamburg, Germany	aqua, hydrated silica, sorbitol, hydroxyethylcellulose, olaflur (1.400 ppm), anethole, carvone, eucalyptol, limonene, mentha arvensis extract, mentha piperita (peppermint) oil, mentha viridis (spearmint) leaf oil, menthol, saccharin, limonene, CI 77,891
Ajona	Dr. Rudolf Liebe Nachf. GmbH & Co. KG, Leinfelden-Echterdingen, Germany	calcium carbonate, sodium bicarbonate, sodium lauryl sulfate, bisabolol, saccharin, urea, diammonium phosphate, algin, tricalcium citrate, glycerin, aqua, anethole, citronellol, eucalyptol, eugenol, geraniol, linalool, mentha viridis leaf oil, menthol, thymol, pelargonium graveolens, flower oil

The efficacy of Karex with regard to the remineralization of artificial acid-induced demineralization has only been investigated in one in vitro study [15]. In that study, remineralization was tested in comparison to a fluoride-containing toothpaste as well as to a toothpaste containing bioactive glasses. A fluoride-containing toothpaste (Elmex; GABA, Hamburg, Germany; 1,400 ppm amine fluoride), showed the significantly highest remineralization rates, whereas Karex had the lowest remineralization of all samples [15]. However, it must be noted that this study was conducted on bovine teeth. Results on human enamel are missing so far. Since the data on Karex are insufficient to date, the aim of this in vitro study was to determine whether the HAP-containing toothpaste Karex is as effective as Elmex with 1,400 ppm amine fluoride in promoting remineralization and preventing afresh demineralization. A HAP- and fluoride-free toothpaste (Ajona; Liebe, Leinfelden-Echterdingen, Germany) served as a control.

The null-hypothesis was that the HAP-containing toothpaste Karex would remineralize in vitro demineralized enamel as effectively as a fluoride-containing toothpaste and also protect enamel from afresh demineralization.

## Methods

Three different toothpastes were investigated with regard to their remineralization properties of artificially demineralized human enamel:

- Karex (Kurt Wolff, Bielefeld, Germany)
- Elmex (GABA, Hamburg, Germany)
- Ajona (Liebe, Leinfelden-Echterdingen, Germany)

Karex is a fluoride-free toothpaste with the active ingredient HAP. Elmex is a fluoride-containing toothpaste with amine fluoride. Ajona is a toothpaste that contains neither fluoride nor HAP and is therefore considered a control toothpaste (see Table 1).

### Preparation of the samples

For the present in vitro study, 19 intact human wisdom teeth were used, which were surgically removed before tooth eruption. Surgical removal of all teeth was performed at the Department of Oral and Maxillofacial Surgery at Münster University Hospital (Germany). All patients were of legal age, were fully informed about the planned study, and gave written informed consent to participate. All human specimens were handled strictly according to the “Declaration of Helsinki” (Local ethics protocol code 2021‐608‐f‐N).

Immediately after surgery, teeth were stored for one hour in a 0.05% thymol solution for disinfection, rinsed thoroughly with distilled water, and stored in distilled water at 5 °C in a refrigerator until further processing.

To define exactly reproducible enamel surfaces on the extracted teeth, 3 vestibular and 4 oral grooves were made in the mesio-distal direction with a diamond cut-off wheel (946; shank 104-HP, size 220, Brasseler, Lemgo, Germany). Subsequently, a further groove was created vestibular-cervically with a surgical round-headed rotating instrument (H71; socket 104-HP, size 008, Brasseler). This defined 4 enamel surfaces next to each other on each tooth, which could be used for demineralization and remineralization and will be referred to as analysis surfaces in the following. The analysis surfaces had a size of 2 mm in width and 8 mm in length. In addition, there were two control surfaces, each of which was located between the analysis surfaces. The control surfaces had a size of 1 mm in width and 8 mm in length.

**Fig. 1:**
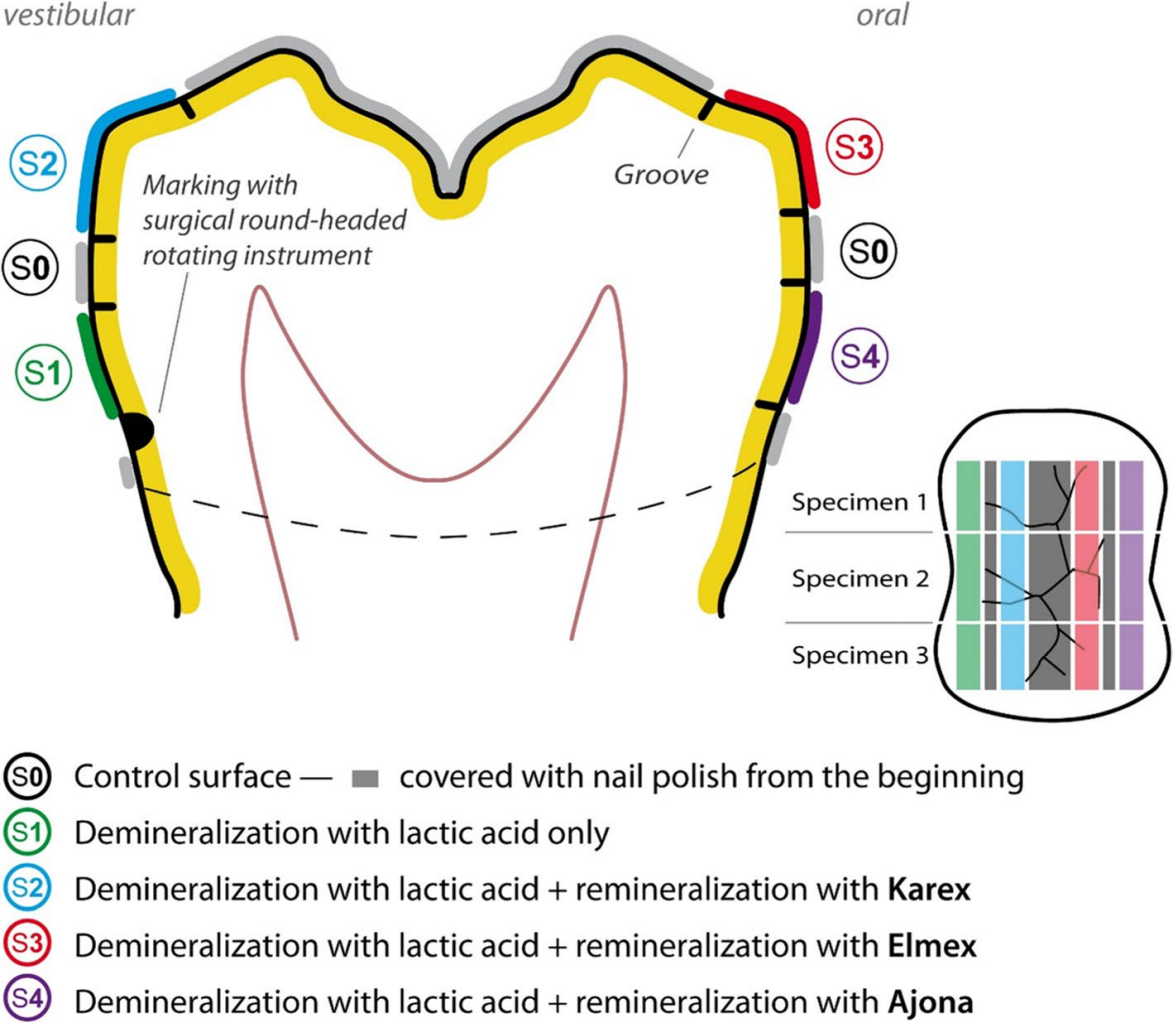
Systematic drawing of how the respective enamel surfaces on a tooth were demineralized and remineralized with the corresponding toothpastes in order to obtain the individual samples for SEM evaluation. (large image: cross-section with surfaces S0—S4, small image: schematic view from occlusal with vertical cuts to obtain the SEM specimens.)

The two control surfaces and the outer marginal occlusal and cervical areas were covered with clear nail varnish (Superstay Forever Strong 7 Days crystal clear 25; Maybelline, New York, USA) and dried for 60 s. The surfaces covered with nail varnish (control surfaces) were thus protected from contact with acid and toothpaste (chemically and mechanically) during the further course of the test. (Fig. 1) Accordingly, neither de- nor remineralization processes took place on these surfaces. This was verified in preliminary tests.

### Demineralization of the samples

All teeth were then placed in 90% lactic acid (pH 3; Pharmacy of the University Hospital, Münster, Germany). After 8 h, the teeth were removed from the lactic acid, rinsed with distilled water (Pharmacy of the University Hospital) and dried.

The following analysis surfaces (S) were defined on the teeth in each case.

- S0: control surface
- S1: demineralization with lactic acid only
- S2: demineralization with lactic acid + remineralization with Karex
- S3: demineralization with lactic acid + remineralization with Elmex
- S4: demineralization with lactic acid + remineralization with Ajona

### Remineralization of the samples

For the examination of Karex, the first analysis surface S1 was covered with clear nail varnish after acid treatment. In contrast, the analysis surfaces S3 and S4 were reliably protected from access of toothpaste, and thus mechanical and/or chemical influences, with liquid rubber dam (Easydam; DeltaMed, Friedberg, Germany). Subsequently, Karex was applied to the analysis surface S2 and brushed for 15 s with a manual toothbrush (Sensitive Super Soft; Dontodent, Karlsruhe, Germany) at a brushing load of 200 g. The brushing load was checked in a preliminary test using a scale. Afterwards, Karex remained on the tooth surface for further 105 s, resulting in a total contact time of 2 min. After thorough rinsing of the tooth with distilled water and appropriate drying, the analysis surface S2 was covered with clear nail varnish and thus fixed.

Easydam was then carefully removed from the analysis surface S3 using a scalpel. In analogy to the treatment with Karex, S3 was brushed with Elmex and a manual toothbrush for 15 s with circular movements at a brushing load of 200 g. The toothpaste remained on the tooth for further 105 s. After rinsing with distilled water and drying, the analysis surface S3 was also covered with clear nail varnish and fixed. Easydam was then carefully removed from analysis surface S4 using a scalpel. In analogy to the treatment with Karex and Elmex, S4 was brushed with Ajona and a manual toothbrush for 15 s with circular movements at a brushing load of 200 g. The toothpaste remained on the tooth for further 105 s. After rinsing with distilled water and drying, the analysis surface S4 was covered with clear nail varnish and fixed.

### Scanning electron microscope evaluation

For further scanning electron microscopy (SEM) analysis of the surfaces, the 19 teeth were cut using a saw (WOCO 50; Uniprec, Clausthal-Zellerfeld, Germany). For this purpose, the clinical tooth crown was first separated from the root by a horizontal cut at the enamel-cement junction. Vertical cuts in the vestibular-oral and occlusal-cervical directions, respectively, were used to obtain three specimens from one tooth, resulting in a total of 57 specimens. The cuts were made perpendicular to the demineralized surfaces in identical distances. These 57 specimens were degreased with acetone (density 0.792 g/cm^3^; Meffert, Bremgarten, Switzerland) and dried for 24 h in a drying oven (T5028; Heraeus, Hannover, Germany). This was followed by embedding in acrylic (TransOptic Compression Mounting Compound; Buehler, Lake Bluff, USA). The resulting specimens were smoothed with diamond-coated abrasive paper (18 μm grit; Waterproof Silicon Carbide Paper, Struers, Willich, Germany) in a grinder (MetaServ 250 Grinder-Polisher; Buehler, Lake Bluff, USA), sprayed with diamond spray (DP-Spray P; Struers, Willich, Germany), and polished to a mirror finish in a polishing machine (PM5; Logitech, Glasgow, United Kingdom). The finished samples were then coated with carbon 25 μm thick in a carbon coater (Carbon coater 208 carbon, Cressington, Dortmund, Germany).

**Fig. 2:**
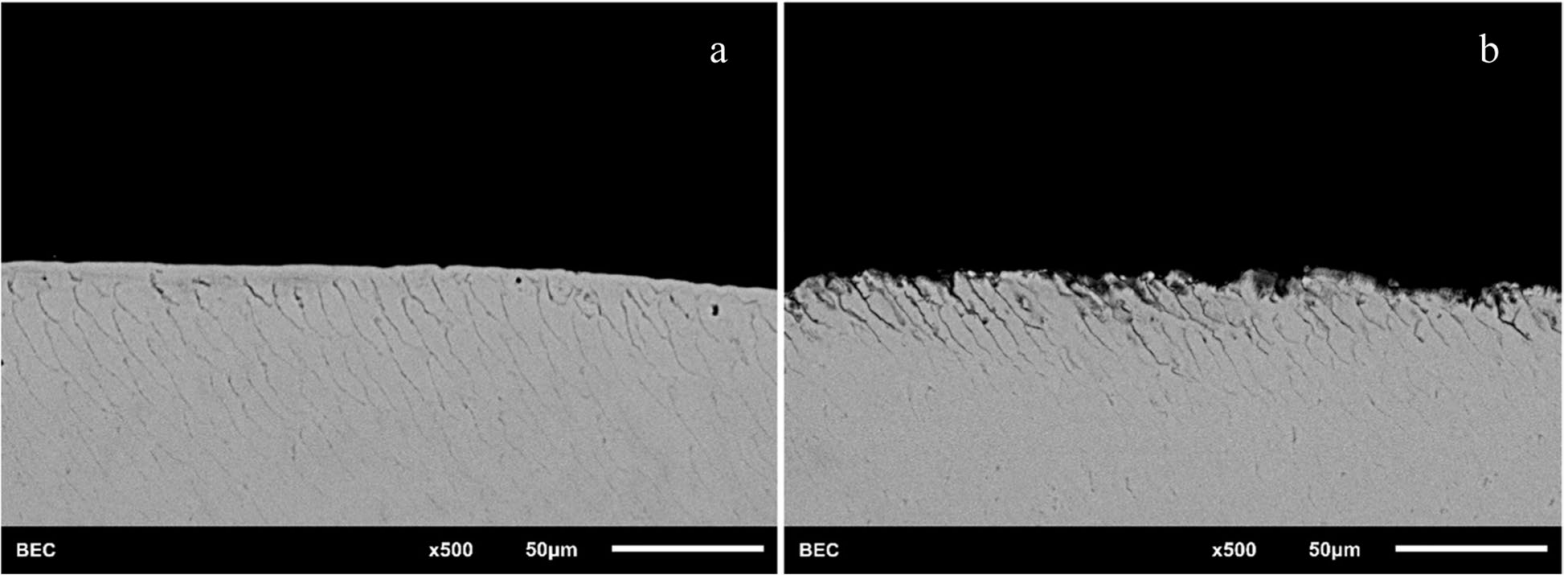
SEM images of a representative specimen before and after demineralization of 8 h. Grey areas represent the intact enamel (enamel prism) and the black areas the defective enamel (corresponding to demineralization by the lactic acid). (**a** = control group without treatment; **b** = surface after 8 h demineralization with lactic acid)

**Fig. 3:**
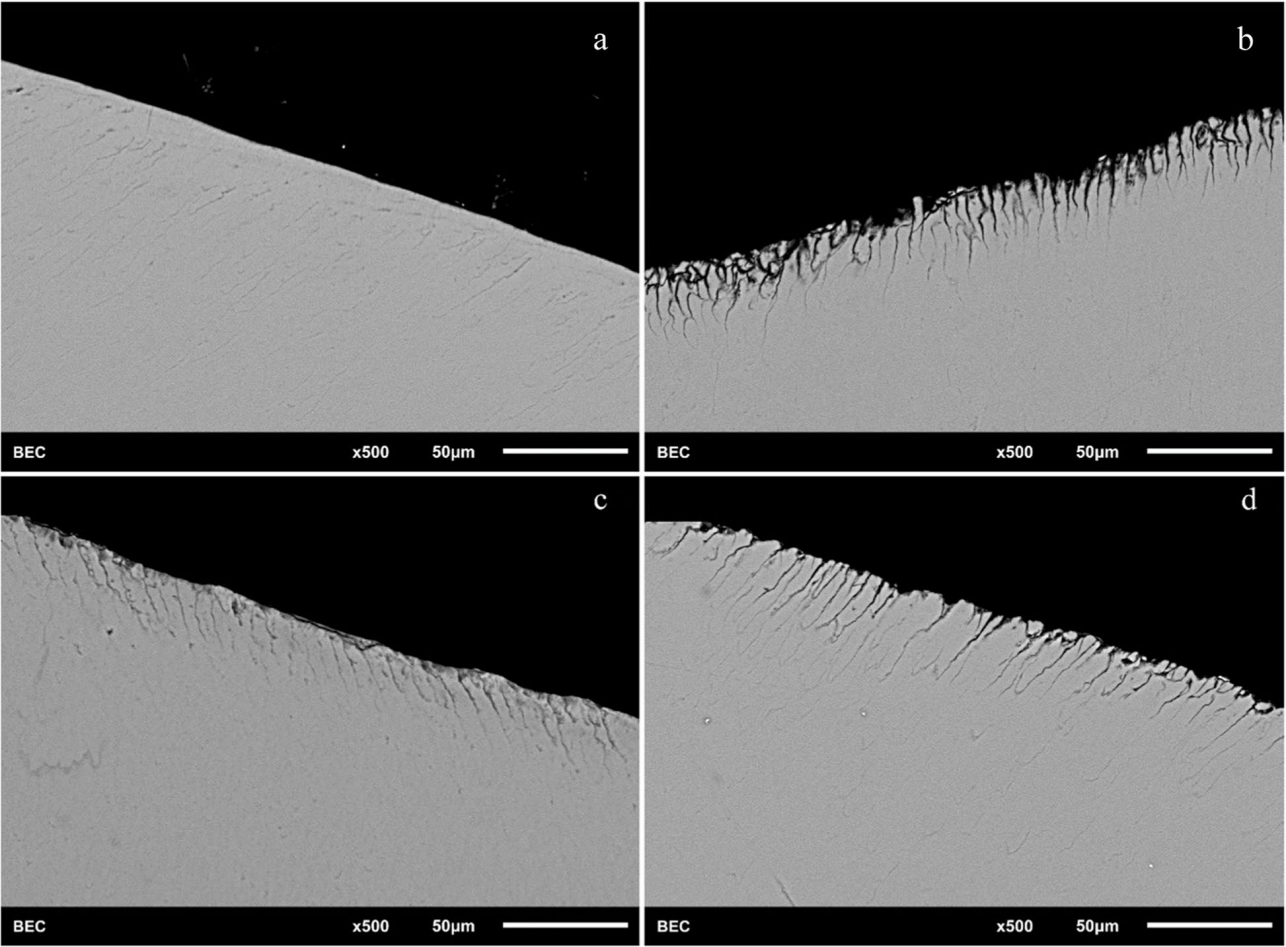
SEM images of a representative specimen in the different toothpaste groups after demineralization of 8 h and remineralization (brushing with the appropriate toothpastes). Grey areas represent the intact enamel (enamel prism) and the black areas the defective enamel (corresponding to demineralization by the lactic acid). (**a** = control group without treatment; **b** = Karex; **c** = Elmex; **d** = Ajona)

The four analysis surfaces were photographed at different magnifications (150 × , 300 × , 500 × , and 1000 ×) using a scanning electron microscope (JSM-6510LV, JEOL, Zurich, Switzerland). In the next step, the 300 × magnification images were transferred to imaging software (ImageJ; Wayne Rasband, NIH, Bethesda, USA) and subjected to pixel analysis. The images of 300 × magnification proved to be particularly suitable in preliminary tests, since here the largest possible analysis area was given with sufficient visibility of the fusion prisms within the image section. Each image was inserted individually into ImageJ and first calibrated using uniform scale at a height of 50 μm in the image. The image was then rotated so that the enamel surface was aligned parallel or perpendicular to the image frame. A “region of interest” was determined by placing a rectangle along the enamel surface. The depth of the rectangle was fixed at 100 μm. The length of the rectangle was chosen to be as large as possible to define the largest representative area. Finally, the Crop function was used to crop out the “region of interest” from the original image. Using the Threshold function, the percentage of demineralization was calculated in the precisely defined, reproducible image section. For this purpose, a black and white image was generated in which the grey pixels represent the intact enamel (enamel prisms) and the black pixels represent the defective enamel (corresponding to demineralization by the lactic acid). The higher the percentage grey, the better the remineralization and the lower the demineralization. The higher the percentage black, the more demineralization and less remineralization. (Figs. 2 and  3).

### Further demineralization of the samples

**Fig. 4:**
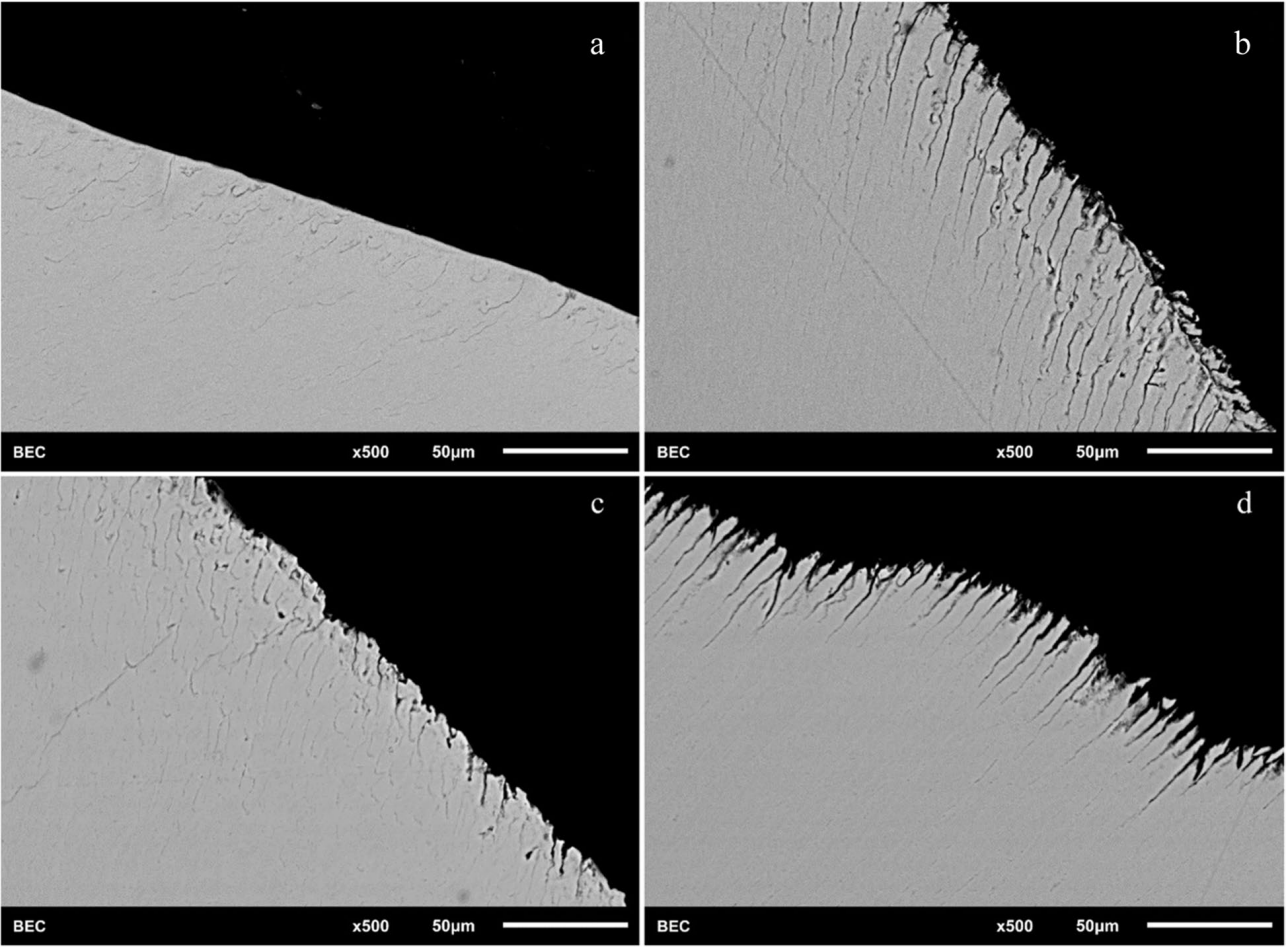
SEM images of a representative specimen in the different toothpaste groups after demineralization of 8 h, remineralization (brushing with the appropriate toothpastes) and new demineralization of 2 h. Grey areas represent the intact enamel (enamel prism) and the black areas the defective enamel (corresponding to demineralization by the lactic acid). (**a** = control group without treatment; **b** = Karex; **c** = Elmex; **d** = Ajona)

In order to check whether the treatment of the enamel specimens with the respective toothpaste protects against a new acid attack, the tooth specimens that have been already evaluated in the SEM were bedded out of the acrylic with the aid of acetone and thoroughly rinsed with distilled water. Afterwards, all tooth specimens were again immersed in 90% lactic acid (pH 3) for 2 h, thoroughly rinsed with distilled water, re-embedded in acrylic and prepared for SEM analysis in analogy to the first series of experiments. An identical analysis to the first series of experiments was performed using SEM. (Fig. 4).

A total of 513 images were analyzed. Of these, 5 different surfaces were analyzed in the 1st test series with 57 images each and 4 different surfaces (minus the control surface) with 57 images each in the 2nd test series. All images generated by ImageJ (*n* = 513) were evaluated independently by two expert observers (LG, AR). Only minor differences were found. In case of discrepancies (*n* = 15 ≙ 2.9%), the affected images were evaluated by another independent expert observer (TD) in a third round. All participants were previously calibrated for evaluation. All samples were blinded, i.e. the evaluators did not know how the samples had been treated beforehand.

### Statistical evaluation

Data were analysed using SPSS software (IBM SPSS Statistics 27, Armonk, USA). Distribution of the data was evaluated using the Kolmogorov–Smirnov test. Normally distributed data (demineralization within the previously defined image section of the respective analysis areas S0 to S4) were further analysed using ANOVA (Analysis of Variance) with post-hoc Scheffé test. For analysis of non-normally distributed data, the Kruskal–Wallis test was applied. Pairwise comparisons were performed using the Mann–Whitney U-test. The level of significance was set at *p* < 0.05.

## Results

### First test series (remineralization)

The descriptive results for the first series of experiments (V1) founder given in Table 2. The results of the ANOVA analysis showed that the mean values between the purely demineralized samples (S1) at the level of 11.78% and all the analysis surfaces brushed with toothpaste (S2, S3, S4) at the level of 7.92% were highly significantly different from each other (*p* < 0.001). All toothpastes resulted in remineralization of demineralized enamel.

**Table 2:** Descriptive statistics of the first test series. The values show the percentage of demineralization (= black pixels) in a defined image section of the respective analysis surfaces (S0—S4) after 8 h of demineralization and 2 min of remineralization with the different toothpastes (Karex, Elmex and Ajona)

Surface	Number	Mean (%)	Standard Deviation (%)	Min/Max (%)
S0 = control surface	57	0.35 ^**a**^	0.24	0.04/0.84
S1 = only demineralization with lactic acid	57	11.78 ^**b**^	1.26	10.02/13.81
S2 = demineralization with lactic acid + remineralization with Karex	57	9.85 ^**c**^	1.63	7.41/12.71
S3 = demineralization with lactic acid + remineralization with Elmex	57	5.01 ^**d**^	0.98	3.58/6.98
S4 = demineralization with lactic acid + remineralization with Ajona	57	8.89 ^**e**^	1.41	6.20/11.93

Values with different superscript letters were statistically different at *p* < 0.001 (*ANOVA*)

A pairwise comparison (Scheffé test) showed that the mean values of the toothpaste-treated analysis surfaces (S2—S4) also differed significantly from each other (*p* < 0.001). Thus, the analysis surfaces treated with Elmex (S3) had a mean value of 5.01% (corresponding to the mean percentage of demineralization). The analysis surfaces treated with Ajona (S4) displayed a mean value of 8.89%; Karex showed a value of 9.85% (S2). The mean value of the percentage of demineralization of the surfaces treated purely with lactic acid (S1) was 11.78%. The control surfaces (S0) showed an average value of 0.35%. Hence, Elmex (S3) exhibited the lowest demineralization and thus remineralized most effectively, followed by Ajona (S4) and Karex (S2). Thus, Karex remineralized highly significantly less compared to the other toothpastes tested (*p* < 0.001).

### Second test series (new demineralization)

Due to the non-normally distributed data, the medians of the percentage of demineralization of the previously defined image section of the respective analysis surfaces (S1, S2, S3, S4) were compared with each other for the second test series (V2). The corresponding descriptive results are listed in Table 3. The Kruskal–Wallis test revealed a highly significant difference between the different toothpastes of V2 (*p* < 0.001). Elmex (S3) showed the best protection against a new demineralization with a median of 6.29%, followed by Ajona with a median of 11.90%. The worst result was obtained for Karex with a median of 13.46%. Consequently, compared to Ajona and Elmex, Karex protected highly significantly worse against a new acid attack after brushing (*p* < 0.001).

**Table 3:** Descriptive statistics of the second test series. The values show the percentage of demineralization (= black pixels) in a defined image section of the respective analysis surfaces (S0—S4) after first 8 h of demineralization and subsequent remineralization for 2 min with the different toothpastes (Karex, Elmex and Ajona) as well as final 2 h demineralization

Surface	Number	Median (%)	25^th^ percentile (%)	75^th^ percentile (%)	Min/Max (%)
S1 = only demineralization with lactic acid	57	14.87 ^**a**^	13.90	15.70	11.44/18.37
S2 = demineralization with lactic acid + remineralization with Karex	57	13.46 ^**b**^	12.10	14.80	9.94/16.76
S3 = demineralization with lactic acid + remineralization with Elmex	57	6.29 ^**c**^	5.63	7.34	3.91/8.87
S4 = demineralization with lactic acid + remineralization with Ajona	57	11.90 ^**d**^	10.30	13.40	8.49/14.99

Values with different superscript letters were statistically different at *p* < 0.001 (*Kruskal–Wallis test*)

The results of the Mann–Whitney U test showed a highly significant difference between the samples demineralized for the second time (V2) and the toothpaste samples from V1 for all analysed surfaces (*p* < 0.001) (Table 4). The difference in the medians between test series V2 and test series V1 was 1.42% for Elmex, 3.24% for Ajona and 3.57% for Karex, respectively. Consequently, Karex showed a highly significant greater enamel loss after a new demineralization and a lower remineralization potential than Ajona and Elmex (*p* < 0.001).

**Table 4:** Results of the comparison of the analysis plots (S1—S4) from test series 1 (V1) with the corresponding analysis plots from test series 2 (V2)—Mann–Whitney U test

Surface	Median V1 (%)	Median V2 (%)	Difference of the medians (V2-V1) (%)
S1 = only demineralization with lactic acid	11.86	14.87	3.01 ^**a**^
S2 = demineralization with lactic acid + remineralization with Karex	9.89	13.46	3.57 ^**b**^
S3 = demineralization with lactic acid + remineralization with Elmex	4.87	6.29	1.42 ^**c**^
S4 = demineralization with lactic acid + remineralization with Ajona	8.66	11.90	3.24 ^**d**^

Values with different superscript letters were statistically different at *p* < 0.001 (*Mann–Whitney U test*)

## Discussion

The aim of this in vitro study was to assess the ability of the HAP-containing toothpaste Karex to remineralize enamel and to protect it from a new demineralization. According to our knowledge, this is the first study on Karex conducted on human enamel. The present results reveal that the HAP-containing toothpaste Karex had a highly significant lower remineralization potential compared to the fluoride-containing toothpaste Elmex. Furthermore, enamel loss after remineralization was significantly higher in teeth samples previously brushed with Karex. The HAP- and fluoride-free toothpaste Ajona served as control group. According to the manufacturer, Ajona is supposed to promote the remineralization of the enamel through a high content of calcium and phosphate ions and thus harden the tooth structure. However, scientific studies supporting this assertion are lacking. In the present study, Ajona showed better remineralization potential than the HAP toothpaste Karex. Thus, the null-hypothesis had to be rejected.

### Discussion of the method

Wisdom teeth that had not yet been exposed in the oral cavity were used for the study. The teeth had therefore never been in contact with any oral hygiene products that might contain fluoride. This ensured that the enamel had not yet been locally remineralized, which might otherwise have led to a falsification of the results. In the case of teeth that had already been exposed to the oral cavity, it remains unclear which de- and remineralization processes have taken place on the enamel surface. By using impacted wisdom teeth, it can be ensured that no alterations have occurred in the original enamel structure.

In addition, all three toothpastes were always tested on one and the same impacted wisdom tooth, so that a direct comparison of the surfaces under the scanning electron microscope was possible. Thus, three enamel samples always belong to one wisdom tooth, which meant that three analysis surfaces per toothpaste could be compared.

In the present study, the toothbrush was used with a contact pressure of 200 g. In literature, the values of the simulated brush load vary between 1 N (100 g) and 3 N (300 g). We therefore based the brush load on an average value of 200 g (2 N), which was also chosen in another comparable study [16].

SEM was chosen as method for evaluation of the de- and remineralization effects. Vertical cutting and SEM evaluation allowed evaluation of not only de- and remineralization effects on the enamel surface, but also in the depth of enamel samples. To the best of our knowledge this is the first kind of such study where SEM visualization was chosen, to analyse the depth of demineralization in enamel samples by imaging software. In several other studies e.g. Vickers hardness test, microradiography, micro-CT, confocal laser scanning microscopic evaluation (CLSM), transverse microradiography (TMR) or polarized light microscopy were used (alone or in combination). Preliminary tests, however, showed that SEM evaluation led to the most reproducible results with the experimental setup chosen here.

In vitro tests have the advantage that they can be carried out under well-controlled experimental conditions. It is important that a de- and remineralization model should always be used to test caries- and erosion-preventive effects, in which acid attacks, applications of the substance to be tested for remineralization and a new acid attack should alternate. Only in this way can statements be made about the remineralizing potential of a substance. However, the complex conditions of the oral cavity can only be simulated to a very limited extent [12, 15]. A disadvantage of the present study may therefore be that the influence of microorganisms and biofilm or plaque (caries model) could not be tested. Also, other modifying factors such as pellicle formation, bacteria and fluoride in saliva and sulcus fluid were not considered in this study. In addition, it should be noted that the study design does not adequately reflect the dynamic process of caries progression with alternating phases of de- and remineralization compared to a pH-cycle model. Therefore, this study can provide information on the remineralization potential of the products used, but not on caries-inhibiting properties [15].

Storage of samples in isotonic saline solution between tooth brushing periods is not able to imitate intraoral mineralization processes [17]. Furthermore, it should also be considered that preparations that support remineralization under laboratory conditions when the tooth surface is clean do not necessarily have a caries-preventive effect [12]. It is just that this has been adequately and evidence-based demonstrated for fluoride-containing oral care products [2–5].

The remineralization of enamel surfaces after acid etching in vitro is likely more pronounced than in vivo. Some proteins found in saliva, such as statherine, PRP (= proline-rich phosphorus), cystatins, or albumins, have a high affinity for HAP and can thus inhibit (re)mineralization [18, 19]. However, since these proteins hardly penetrate into an initial lesion due to their size, remineralization can take place in initial carious lesions with the help of fluoride [20]. In contrast, in non-carious enamel lesions created directly with an acid, the defects are larger in diameter, allowing these salivary proteins to penetrate the enamel surface and thus inhibit remineralization. For example, it has been shown that enamel etched with acid retains its etching pattern in the oral cavity for months because no remineralization occurred due to the incorporation of the proteins [21].

Since products that claim to enable remineralization should also be able to induce remineralization in an in vitro model, this study should be considered as a first investigation in this regard. However, it was also not the aim of this study to simulate cariogenic processes of the oral cavity. Rather, the sole aim was to investigate the pure remineralization capacity of the toothpastes and a possible protection against renewed demineralization, deliberately without including other factors.

## Discussion of the results

Similar to the enamel, calcium/phosphate precipitates are relatively easily soluble in acid, so it can be assumed that such mineral precipitates may have only limited, if any, protective effects [22]. Accordingly, the experiments that have investigated the effects of HAP in a cyclic erosion model have shown little or no effects of HAP formulations [16, 22, 23].

There may be differences in the mechanism of action related to the size of the HAP particles [24]. Clinical efficacy has been found to depend on the specific geometry, pore size, and degradation rate of the HAP, with a smaller particle size being beneficial [25].

An in vitro study showed that nano-HAP enabled the remineralization of demineralized enamel, while no difference was observed between pure water and micro-HAP [26]. The Karex toothpaste investigated in the present study contains 10% micro-HAP [12, 15]. It can be assumed that the micro-HAP particles are less effective in penetrating the lesion body of the demineralized enamel. This would explain the lowest measured remineralization potential in this study, which was even lower than in the control group in which a fluoride- and HAP-free toothpaste was used.

However, the results of this study on Karex (micro-HAP) coincide with the results of an in vitro study on a nano-HAP-containing toothpaste (Biorepair; Kurt Wolff, Bielefeld, Germany) [27]. The investigated nano-HAP toothpaste (Biorepair) showed no effect; only the use of a fluoride-containing toothpastes resulted in a reduction of demineralization [27]. Also, 10% and 20% experimental nano-HAP toothpastes were both unable to reduce enamel demineralization, in contrast to a fluoride-containing toothpaste [28].

The HAP crystals in toothpastes may not seal the small defects of incipient enamel caries, but erode them more than the other abrasive agents. It has therefore been speculated that the significantly lower incidence of enamel caries after daily brushing with toothpaste containing HAP in vivo may be due to the abrasive loss of early caries lesions [29]. Due to this increased abrasiveness, a misinterpretation is possible in the selected in vitro test protocol of the present study, i.e. the more abrasive toothpaste would simulate a higher “remineralization”, since demineralized enamel would be most effectively eroded by the abrasive toothpaste. According to the manufacturer, Karex has the highest RDA value of the toothpastes tested here, at 60. The RDA value of Elmex is between 50 and 60; the RDA value of Ajona is 30. If the RDA value had had an influence on “remineralization” in the test protocol selected here, Karex would therefore have to enable the best remineralization. However, this is not the case. Karex exhibits the significantly lowest remineralization rate. An influence of the RDA value by abrasion of the demineralized enamel on the results presented here can therefore not be determined.

In addition to HAP, Karex also contains zinc and xylitol. Both ingredients are known to have antimicrobial effects in toothpaste and to prevent the formation of dental plaque. It can therefore also be speculated that the possibly observed caries-protective effect of Karex [14] may be due to zinc and xylitol and not solely to the HAP.

Another possible influencing factor on the remineralization potential of toothpastes could be their pH value. For example, Karex has a saliva-neutral pH, whereas the pH of Elmex is in the acidic range. It is assumed that a product with an acidic pH remineralizes better than a product with a neutral pH. Several studies have shown a reinforcing effect for fluoride-containing agents by lowering their pH, which is associated with an improved ability to absorb minerals into enamel defects [30–32].

New agents should represent an improvement over established agents already in use that are easy to use, inexpensive, and safe. For caries and erosion prevention, such agents are available in the form of the various fluoride compounds. This means that a new active ingredient should surpass the effect of fluorides [12]. This could not be demonstrated for Karex in this in vitro study.

## Conclusions

Under the conditions of this in vitro study, a fluoride-containing toothpaste (Elmex) was significantly superior in comparison to a hydroxyapatite-containing (Karex) and a fluoride- and hydroxyapatite-free toothpaste (Ajona) with regard to remineralization potential as well as the extent of protection against renewed demineralization of enamel. The recommendation to use Karex to protect enamel against demineralization should be critically questioned.

## Data Availability

The datasets used and/or analysed during the current study are available from the corresponding author on reasonable request.

## Abbreviations

HAP: Hydroxyapatite
PRP: Proline-rich phosphorus
S: Analysis surfaces
SEM: Scanning electron microscopy
V1: First series of experiments
V2: Second series of experiments
